# Stress Marks on the Genome: Use or Lose?

**DOI:** 10.3390/ijms20020364

**Published:** 2019-01-16

**Authors:** Bayan Bokhari, Sudha Sharma

**Affiliations:** 1 Department of Biochemistry and Molecular Biology, College of Medicine, Howard University, 520 W Street, NW, Washington, DC 20059, USA; bayan.bokhari@bison.howard.edu; 2 Department of Biochemistry, Faculty of Applied Medical Science, Umm Al- Qura University, Makkah 21421, Saudi Arabia; 3 National Human Genome Center, College of Medicine, Howard University, 2041 Georgia Avenue, NW, Washington, DC 20060, USA

**Keywords:** oxidative stress, DNA damage, DNA repair, replication, 8-oxoG, epigenetic, gene expression, helicase

## Abstract

Oxidative stress and the resulting damage to DNA are inevitable consequence of endogenous physiological processes further amplified by cellular responses to environmental exposures. If left unrepaired, oxidative DNA lesions can block essential processes such as transcription and replication or can induce mutations. Emerging data also indicate that oxidative base modifications such as 8-oxoG in gene promoters may serve as epigenetic marks, and/or provide a platform for coordination of the initial steps of DNA repair and the assembly of the transcriptional machinery to launch adequate gene expression alterations. Here, we briefly review the current understanding of oxidative lesions in genome stability maintenance and regulation of basal and inducible transcription.

## 1. Introduction

Millions of years ago the evolution of photosynthesis provided oxygen to our planet Earth. Subsequently living organisms not only have used oxygen in their energy production and metabolism but also developed several protective systems in which they can deal with toxicity generated from reactive oxygen species (ROS). To maintain a healthy status, oxidants and antioxidants should be in equilibrium. The imbalance between oxidants production and detoxification causes oxidative stress which is the precursor to oxidative damage to proteins, lipids, and DNA compromising their structure and functions. This in turn can impair normal physiological functions and lead to a variety of diseases and aging [1,2,3].

Oxidative damage to DNA is especially problematic since DNA cannot be resynthesized or turned over. Reactive oxygen species, which include reagents such as superoxide anions (O_2_^•−^), hydrogen peroxide (H_2_O_2_), and hydroxyl radicals (^•^OH) can be produced from oxidative metabolism in mitochondria and other endogenous sources such as peroxisomes and inflammatory cells [3]. Many environmental factors have been identified as exogenous sources for ROS initiation, including but not limited to exposures to chemicals like bisphenol A or toxins like organophosphate insecticides, ultraviolet, and ionizing radiations [1]. The ROS can attach to DNA due to higher reactivity with strong nucleophilic sites on nucleobases. A variety of mutagenic products, such as base modifications or base transversions can be generated through the reactions with either the DNA bases or the deoxyribose sugars [1]. Furthermore, oxidative damage to DNA may lead to mutations that activate oncogenes or inactivate tumor suppressor genes as well as modification of gene expression [3].

Sequence characteristics of the DNA render certain regions of the genome more susceptible to oxidative stress [4,5]. Mitochondrial DNA (mtDNA) is more accessible to free radical injury owing to its proximity to the site of O_2_^•−^ generation from the electron transport chain [6]. The production of ROS by mitochondria leads to mtDNA damage and mutations which in turn lead to progressive mitochondrial dysfunction and to a further increase in ROS production [7,8]. The absence of histone protection and availability of fewer repair mechanism also makes mtDNA more susceptible to ROS damage than the nuclear genome [6,9]. As an abundant endogenous source of DNA damage, ROS-induced stress is widely attributed to promote catastrophic consequences for aging [2] and related diseases such as cancer [10] and neurodegeneration [11].

**Figure 1:**
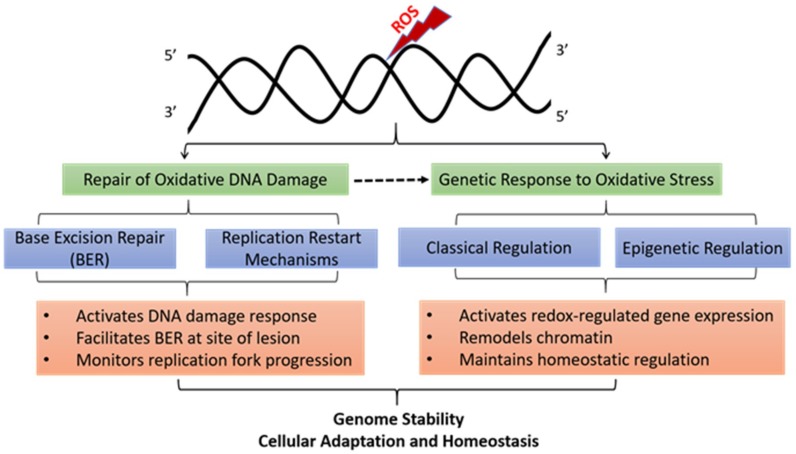
Mechanisms of oxidative-stress-induced genetic and epigenetic alterations. Damage to DNA bases due to oxidative stress induced from the plethora of extracellular and intracellular factors is deleterious, leading to stalled replication forks and mutations. Mammalian cells utilize the base excision repair (BER) pathway alone and in concert with various replication restart mechanisms to get rid of oxidative lesions and ensure faithful duplication of genome. Genetic response to oxidative stress involves alteration in gene expression by both the classical gene regulatory mechanisms and by epigenetic processes. Classical gene regulation implicates transcription-factor based gene regulation to influence gene transcription. Epigenetic mechanisms are those that do not involve changes in the genome sequence, but rather in nuclear architecture, chromosome conformation, and histone and DNA modifications. For example, epigenetic involvement of ROS has been attributed to oxidative conversion of 5-mC to 5-hmC. Oxidative conversion of G to 8-oxoG at the promoter regions activates expression of redox-regulated genes suggesting that oxidative base modification may also represent an epigenetic mark serving as sensors of oxidative stress. Involvement of DNA repair (largely BER) in coordinating the gene regulatory response to oxidative stress is indicated by dashed arrow.

Here we review how oxidative stress challenges the duplication and transmission of genetic information by causing direct DNA damage, regulating the activity of DNA repair enzymes, and altering basal and inducible transcription (Figure 1). We also discuss the epigenetic role of oxidative base modifications in coordinating DNA repair and adequate gene expression changes following oxidative stress.

## 2. Specific Oxidative Base Modifications

More than a hundred different types of base damage have been identified as products of oxidative stress [12]. All four DNA nucleobases are susceptible to damage by ROS leading to modification of their structure and alteration of the base-pairing properties (Table 1).

Guanine (G) is the most frequently oxidized base due to its low oxidation potential. As a result, 8-oxo-7,8-dihydro-2′-deoxyguanosine (8-oxoG) is the most abundant oxidative DNA lesion which is moderately mutagenic resulting into G:C to T:A transversion and has been associated with cellular transformation and cancer initiation [13,14]. Structural studies showed that 8-oxoG induces only minor distortions to the DNA helical structure that are localized near the modification site. The base pairing preference is determined by the conformation; anti-8oxoG base pairs with cytosine (C) whereas syn-8-oxoG functionally mimics thymine (T) and base-pairs with adenine (A) thus giving rise to A:8-oxoG mismatches which potentially results in CG→AT transversion mutations [15]. Replicative DNA polymerases are slowed down at 8-oxoG and insert both correct cytosine and incorrect adenine opposite 8-oxoG, but they preferentially extend A:8-oxoG mispairs. However, during replication events, the cells have an opportunity to utilize the translesion synthesis (TLS) polymerases, mainly the Y-family polymerases, for rapid bypass of 8-oxoG lesion to prevent replication fork arrest [16,17]. The 8-oxoG is also highly susceptible to further oxidative damage, yielding the additional mutagenic base lesions spiroiminodihydantoin and guanidinohydantoin. Oxidation of guanine also results in fragmentation of the purine imidazole ring leading to another major oxidative lesion, 2,6-diamino-4-hydroxy-5-formamidopyrimidine (FapyG) [12,13]. Oxidation of adenine can lead to two major products: 8-oxo-7,8-dihydro-2′-deoxyadenosine (8-oxoA) and 4,6-diamino-5-formamidopyrimidine (FapyA). A less prevalent adenine modification upon oxidative damage is 2-hydroxydeoxyadenosine-5′-triphosphate (2OHA) [12,13].

Cytosine can be the target of oxidation only at the 5,6-double bond to form a major oxidative product 5-hydroxy-2′-deoxycytidine (OH5C) which can be found on DNA spontaneously and after exposure to ROS generating chemicals [12,18]. In contrast, a cytosine analogue, methylcytosine (5mC), can be attacked by free radicals at both the 5,6-double bond and the 5-methyl group and several oxidation products of 5mC can be generated [12,18]. Thymine base can also be attacked by free radicals on either the 5,6-double bond or the 5-methyl group, generating various oxidative products. Thymine glycol (Tg) is one of the most examined oxidative products generated by the ring opening on the 5,6-double bond of thymine causing inhibition to replicative polymerases and a mutagenic signature indicative of translesion synthesis [17]. Moreover, thymine can be oxidized to produce the 5,6-dihydrothymine (DHT) which despite being targeted for repair, does not appear to cause mutations or cytotoxicity [18]. The free radical attack on the 5-methyl group of thymine produces numerous oxidation products including 5-hydroxymethyluracil (5hmU) which can base pair with both adenine and guanine, thus leading to T:A→C:G transition [19].

## 3. Repair of Oxidative DNA Damage

As such, cells regularly encounter a spectrum of DNA damage ranging from small non-helix distorting lesions to bulkier adducts that cause significant structural changes to the DNA double helix. Base excision repair (BER) is the main repair pathway of the oxidatively generated 8-oxoG and other non-helix disturbing lesions [14,20,21,22,23]. Base excision repair essentially involves (i) excision of a damaged or inappropriate base by DNA glycosylase, (ii) incision of the phosphodiester backbone by apurinic/apyrimidinic (AP) endonuclease at the resulting abasic site creating a single-strand break (SSB), (iii) termini clean-up to permit unabated repair synthesis and/or nick ligation, (iv) gap-filling to replace the excised nucleotide, and (v) sealing of the final, remaining nick. In addition to BER, other DNA repair pathways including mismatch repair (MMR) and nucleotide excision repair (NER) also contribute to minimize the genotoxic impact of oxidative base lesions as summarized recently [24].

## 4. Oxidative DNA Damage and Replication Stress

The repair of oxidative DNA lesions is essential to avoid stress when cells enter the replicative phase of the cell cycle [25]. To shield the genome from oxidative damage, DNA replication in yeast is restricted to the reductive stage of the metabolic cycle when oxygen consumption is minimal [26]. However, higher eukaryotes must deal with both physiological and pathological levels of ROS while DNA synthesis is ongoing.

Oxidative lesions and BER intermediates interfere with replication, cause single- (SSB) and double-strand breaks (DSB) in DNA, and lead to chromosomal aberrations [27]. Unrepaired SSBs can stall replication machinery which may activate the error-prone damage tolerance mechanism or may lead to fork collapse into a potentially cytotoxic DSB [28]. In addition, closely spaced oxidative lesions, also referred to as oxidative clustered DNA lesions (OCDL), can be converted to DSBs during BER [29].

The single-strand DNA template at the replication fork is more susceptible to oxidative base damage and strand breaks than the nonreplicating DNA [30]. Replication fork progression is blocked by BER-initiating lesions [31] as well as by the DNA structure intermediates arising from the repair of oxidized bases [32]. The G-rich sequences at telomeres [5] and promoters that are known to accumulate oxidative DNA damage also display high rates of replication forks stalling [33]. One of the ways by which cells mitigate negative impact of elevated ROS on the replicating genome is by reducing replication fork speed [34].

Mechanisms that play key roles in the reactivation of arrested replication forks may also act as a barrier against genetic instability triggered by the endogenous oxidative/replication stress axis [34,35]. We have recently demonstrated that RecQ like 1 (RECQL1 or RECQ1) helicase which is critical for resetting of replication fork for resumption of normal DNA synthesis [36] is also important for BER [37]. Using live in cell-repair assays and biochemical reconstitution, we identified that RECQ1 helicase activity and ERCC1-XPF endonuclease in cooperation with poly(ADP-ribose) polymerase (PARP1) and Replication Protein A (RPA) mediate a novel sub-pathway of conventional long-patch BER [37]. This process is facilitated by the well-established interaction among RECQ1, PARP1, and RPA [37,38]. Although RECQ1 modulates cellular response to oxidative stress [38], whether RECQ1 is required to sustain fork progression following oxidative stress is yet unknown. Nevertheless, physical and functional cooperation of DNA replication and BER is emerging as a major regulatory mechanism for preventing genomic instability [39,40].

In addition to inducing DNA damage and nucleotide pool imbalance [35], oxidative stress can alter replication by oxidation induced inactivation of key DNA repair proteins such as RPA [41] or by modulating the levels of Ku70 and Ku80 proteins essential for DSB repair by non-homologous end joining [42]. Oxidative stress leads to activation of the ataxia-telangiectasia mutated (ATM) kinase, the major sensor and regulator of the cellular response to DSBs [43]. Downstream to oxidative stress-dependent activation, ATM protects cells from ROS accumulation by stimulating NADPH production and promoting the synthesis of nucleotides required for the repair of DSBs [44] and a number of other processes to promote restoration of redox homeostasis [45]. Indeed, cells defective in DNA damage response show endogenously elevated levels of ROS [35].

Collectively, these observations emphasize intricate mechanisms that coordinate replication dynamics, activation of DNA damage response and DNA repair as directed by the redox status of the cell.

## 5. Oxidative DNA Damage and Gene Expression Changes

Cellular response to oxidative stress involves highly regulated alteration in gene expression which is shared with gene expression patterns observed in aging [46], cancer [47], and other diseases [48,49]. The in vivo gene expression signature of oxidative stress suggests p53 plays an important role and upregulation of p53 targets genes as a common response to oxidative stress across diverse organs and species [50]. The cellular concentration of ROS appears to influence the selective activation of transcription factors involved in signaling pathways including the nuclear factor erythroid 2-related factor 2 (Nrf2), mitogen-activated protein (MAP) kinase/AP-1, and nuclear factor-kB (NF-kB) pathways, as well as hypoxia-inducible transcription factor 1α (HIF1A) [51]. Oxidative stress and redox signaling may also affect gene expression by altering the functions of histones and DNA modifying enzymes [52].

The presence of 8-oxoG in the template strand would be expected to impair transcription by stalling of RNA pol II [53,54]; however, 8-oxoG in gene promoters is also associated with gene activation [55,56]. Oxidation of bases may serve as critical sensors through which ROS signals are sensed and the transcription from the redox responsive genes is regulated [52,57]. Consistent with this, the increased level of 8-oxoG in the mtDNA of mice lacking 8-oxoguanine DNA glycosylase (OGG1), the enzyme responsible for recognition and repair of 8-oxoG, is associated with differential expression of genes involved in ROS-mediated signaling including pro-inflammatory genes [58]. In another study, ROS generated by tumor necrosis factor alfa (TNFα) exposure of human cells led to OGG1 enrichment primarily at the regulatory regions of a large number of genes constituting signal transduction pathways that modulate redox-regulated metabolic and immune responses for an immediate global cellular response [59]. Studies from various groups have collectively suggested that the redox levels orchestrate OGG1 to play a role either in gene transcription or in lesion repair; and the magnitude of base lesions, predominantly of 8-oxoG, defines the fate of cells [24,60,61,62].

APE1 is another dual function protein involved both in the BER pathways of DNA lesions, acting as the major apurinic/apyrimidinic endonuclease (APE), and in eukaryotic transcriptional regulation of gene expression as a redox co-activator of several transcription factors including AP-1, HIF1-α, and p53 [63]. APE1 plays a role in the regulation of gene expression during oxidative stress condition by interactions via its redox-effecter factor-1 (Ref-1) domain with protein factors such as HIF1-α, STAT3, and CBP/p300 that promote transcription [63]. Another study from Tell lab demonstrated that APE1-dependent and BER-mediated DNA repair promotes the initiation of transcription of sirtuin 1 (*SIRT1*) gene upon oxidative DNA damage [64].

Although the precise mechanisms are yet unclear, recognition and binding of the oxidatively damaged base by the repair proteins during the pre-excision step of BER facilitates the recruitment of specific transcription factors for prompt transcriptional response [61]. Conceivably, oxidative stress-induced gene regulation may act in concert with the repair of DNA damage to protect cells from accumulation of oxidative damage.

## 6. Epigenetic Functions of Oxidative DNA Lesions

Localized formation of 8-oxoG in gene regulatory regions have been suggested to represent an epigenetic modification serving as sensors of oxidative stress. Despite the vulnerability of guanine to oxidation, over 70% of the promoters of human genes, including a large percentage of redox-responsive gene promoters, contain evolutionarily conserved G-rich clusters [65]. Guanine oxidation shows strong distributional bias in the genome with gene promoter and untranslated regions harboring greater 8-oxoG [66].

Oxidative modification of guanine to 8-oxoG in the promoter may provide a platform for the coordination of the initial steps of DNA repair, especially BER, and the assembly of the transcriptional machinery to launch the prompt and preferential expression of redox-regulated genes in cells that are responding to oxidative stress [24,57,67,68]. Indeed, the formation of 8-oxoG in the G-rich promoters of the vascular endothelial growth factor (*VEGF*) [66,69], *TNFα* [70], and *SIRT1* [64] genes can increase transcription via the BER pathway [61,67].

**Figure 2:**
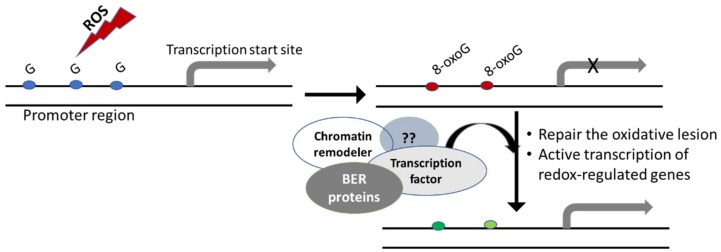
The influence of guanine oxidation at the promoter region on gene expression. Reactive oxygen species (ROS) induces oxidation of guanine to 8-oxoG. Gene promoters are enriched in guanine and sequence motifs prone to form G4 DNA structures. Formation of 8-oxoG is also shown to induce critical topological changes in DNA structure. Binding of 8-oxoG by BER proteins may facilitate the site-specific recruitment of specific transcription factors, chromatin remodelers and other accessory factors (shown as ??). These factors likely work in concert to repair the oxidative base lesion (shown by green) and activate transcription of redox-regulated genes for an adequate cellular response.

Transcription of the *VEGF* gene is known to be regulated by a specific sequence motif in its promoter, called the G4 motif because of its ability to form G-quadruplex (G4) DNA structure [71]. Through complementary biochemical, cellular, and genetic approaches, the Burrows lab demonstrated that the oxidation of guanine to 8-oxoG in the G-rich promoter element of the *VEGF* gene facilitates activation of transcription in a BER-dependent manner since the OGG1-null cells failed to exhibit an increase in gene expression [67,69]. One of the suggested mechanisms is that oxidation of guanine to 8-oxoG in the G4 motif provides a structural switch for recruitment of BER proteins such as APE1 and transcription factors such as HIF1-α to promote gene transcription [67,69]. Similar mechanisms implicating other BER proteins and cooperating factors may operate for transcriptional activation of other redox-regulated genes (Figure 2).

Indeed, the G4 motifs (represented by G_≥3_N_x_G_≥3_N_x_G_≥3_N_x_G_≥3_) are enriched in the promoter regions of many genes [72]. Gene regulation by modulating the topological superstructures of G4 containing promoters, for example *VEGF* as described above and endonuclease III-like protein 1 (*NTHL1*) genes [67], suggest epigenetic role of 8-oxoG modification.

The regulatory and possible epigenetic roles of 8-oxoG in cells that are responding to oxidative stress can be contrasted with a more traditional 5-methylcytosine (5mC) epigenetic modification contributing to the regulation of gene activity during development and differentiation [73,74,75]. Cytosine methylation is generally associated with repressed chromatin and inhibition of gene expression [76,77]. The methyl moiety of 5mC can be eliminated passively during DNA replication, or actively through enzymatic DNA demethylation [78]. Base excision repair is implicated in active demethylation of 5mC in oxidation independent and dependent manner [78]. During active DNA demethylation, for activation of genes silenced by cytosine methylation, the ten-eleven translocation (TET) proteins oxidize 5mC in a stepwise fashion to 5-hydroxymethylcytosine (5hmC), 5-formylcytosine (5fC), and 5-carboxylcytosine (5caC). Both 5fC and 5caC can be recognized and excised from DNA by thymine-DNA glycosylase (TDG) followed by subsequent filling in of unmodified cytosine by the BER pathway [79]. Moreover, passive elimination of 5mC is also enhanced by active DNA demethylation [80].

Oxidative conversion of 5mC to 5hmC under oxidative stress changes the DNA methylation pattern resulting in epigenetic alterations [73]. Enrichment of 5hmC within the gene bodies, promoters, and transcription factor-binding regions suggest it may regulate gene expression by modulating chromatin accessibility of the transcriptional machinery, or by inhibiting repressor binding [73]. Of note, readers of 5hmC include several DNA glycosylases (for example, NEIL1 and NEIL3), replication factors (RFC), helicases (for example, HELLS and RECQ1), and transcriptional repressor protein MeCP2 [76]. MeCP2 recognizes methyl-CpG and recruits co-repressor molecules to silence transcription. Oxidation of guanine to 8-oxoG significantly inhibits MeCP2 DNA binding [81]. Proposedly, OGG1 may alleviate the transcriptional repression by cytosine methylation [61]. By binding to 8-oxoG in the opposite strand, OGG1 may interfere with the interaction of MeCP2 (and other proteins) with their substrates and recruit transcriptional machinery components to activate transcription [61]. Overall this suggests an intertwined and DNA repair-involved DNA demethylation pathway for epigenetic regulation of gene expression.

A recent study suggested that APE1 modulates DNA methyltransferase 1 (DNMT1) expression and consequent promoter methylation in a redox-mediated manner [82]. These observations highlight a strong possibility that oxidative modification to DNA bases, such as in the form of 8-oxoG or oxidized 5mC serve as epigenetic mark and function in a DNA-based mechanism for gene activation.

## 7. Conclusions and Outlook

Cellular redox status strongly impacts genome duplication and transmission. Therefore, it is critical to understand how ROS-induced stress affects replication dynamics and activation of DNA damage response, and how this is coordinated with the transcriptional response for the maintenance of genomic stability and cellular homeostasis.

The impact of oxidative stress on genetic stability through direct damage to the DNA, such as oxidized bases or abasic sites, has been documented extensively. As the primary mechanism counteracting oxidative stress-induced DNA lesions, the BER pathway has been well characterized. Furthermore, activity of BER enzymes such as OGG1 is regulated in a redox-dependent manner [83,84] as well as by posttranslational modifications [85]. The interplay between chromatin status and BER is beginning to be unveiled [86,87], but the molecular mechanisms by which various DNA repair proteins, chromatin remodelers, and transcription factors are targeted to specific oxidative lesions are yet to be delineated. How chromatin remodeling influences BER of oxidative lesions and subsequent gene expression changes remains an exciting open question.

Given the demonstrated role of G4 structures in regulation of redox-sensitive gene expression changes [88], factors that modulate the stability of these structures are expected to play significant roles in the process. Interestingly, binding to G4 motifs in target gene promoters and resolution of G4 DNA structures has been suggested as a mechanism of transcriptional regulation by DNA helicases RECQ1 [89,90], XPB, XPD [91], BLM [92], and WRN [93]. However, in vitro biochemical data suggests that the transcriptional regulation by RECQ1 likely does not involve RECQ1 helicase-mediated unwinding of G4 structures [94]. A potential role of RECQ1 could be to mediate, either directly or through protein–protein interactions, repair of oxidative lesions [37,38] in the G4 motif at promoters and elsewhere and facilitate subsequent alterations in gene expression [89,90]. An important next step in understanding the molecular role of these helicases in the mechanisms of gene regulation is to determine the involvement of cooperating transcriptional partners.

Collective data shows that the repair of oxidative DNA damage, a mechanism that protects genome integrity, also serves as a proactive mechanism to ensure a prompt and adequate transcriptional program as governed by the cellular cues such as redox status [95]. Studies with APE1 and OGG1 suggest that these DNA repair proteins can impact transcription of activator-dependent genes by facilitating DNA repair, chromatin remodeling and assembly of transcriptional machinery at gene promoters, but their roles in constitutive housekeeping transcription is unclear. If 8-oxoG indeed serves as a regulatory mark, epigenetic regulation in this case likely relies on the oxidative DNA damage, possibly induced by the low level of endogenous ROS. If this is the case, then it will be important to determine if the source of ROS, for example endogenous versus exogenous, by which 8-oxoG is introduced in the genome dictates the transcriptional outcome in physiological and pathological states.

How prevalent is 8-oxoG-mediated gene regulation in the mammalian genome is unclear and the epigenetic role of 8-oxoG is yet to be interrogated with respect to biological processes such as differentiation, development, tumorigenesis, and metastasis. If 8-oxoG is indeed a bona fide epigenetic mark, an additional consideration is whether oxidation of guanine to 8-oxoG is an active process. Towards this, targeted generation of 8-oxoG in the promoter regions can be coupled with enzymatically catalyzed oxidative demethylation of histones by the lysine demethylase (LSD1) as has been shown in the estrogen receptor- and MYC-activated gene expression models [96,97]. It would be interesting to determine the relationship between 5mC and 8-oxoG, and the roles of BER and other proteins.

Given the unavoidable exposure to ROS, cells seem to have evolved strategies to utilize ROS as biological stimuli suitable for the physiological need. Oxidative base modifications, therefore, appear to have both beneficial and deleterious functions. While higher levels of oxidative damage might invoke the DNA repair mechanisms to remove the oxidative lesion, lower levels of oxidative damage may serve to regulate gene expression to the degree required to maintain genomic integrity and cellular homeostasis. The mechanism that enables cells to distinguish between “regulatory” oxidative DNA damage from those that cause “undesirable” consequences is yet elusive. Further research is needed to gain a more complete understanding of the molecular details of cellular and genomic context that determine whether to lose or use these stress marks.

## Figures and Tables

**Table 1:** DNA base modifications that commonly exist after oxidative stress.

DNA Base	Oxidized Base Modification
Guanine (G)	8-oxo-7,8-dihydro-2′-deoxyguanosine (8-oxoG) 8-oxoG is further oxidized to: Spiroiminodihydantoin Guanidinohydantoin 2,6-diamino-4-hydroxy-5 formamidopyrimidine (FapyG)
Cytosine (C)	5-hydroxy-2′-deoxycytidine (OH5C)
Adenine (A)	8-oxo-7,8-dihydro-2′-deoxyadenosine (8-oxoA) 4,6-diamino-5-formamidopyrimidine (FapyA) 2-hydroxydeoxyadenosine-5′-triphosphate (2OHA)
Thymine (T)	Thymine glycol (Tg) 5,6-dihydrothymine (DHT) 5-hydroxymethyluracil (5hmU)
